# Evaluation of a Neuromechanical Walking Control Model Using Disturbance Experiments

**DOI:** 10.3389/fncom.2017.00015

**Published:** 2017-03-14

**Authors:** Seungmoon Song, Hartmut Geyer

**Affiliations:** Robotics Institute, Carnegie Mellon UniversityPittsburgh, PA, USA

**Keywords:** neuromechanical simulation, human locomotion, spinal control, model evaluation, spinal reflex, central pattern generator

## Abstract

Neuromechanical simulations have been used to study the spinal control of human locomotion which involves complex mechanical dynamics. So far, most neuromechanical simulation studies have focused on demonstrating the capability of a proposed control model in generating normal walking. As many of these models with competing control hypotheses can generate human-like normal walking behaviors, a more in-depth evaluation is required. Here, we conduct the more in-depth evaluation on a spinal-reflex-based control model using five representative gait disturbances, ranging from electrical stimulation to mechanical perturbation at individual leg joints and at the whole body. The immediate changes in muscle activations of the model are compared to those of humans across different gait phases and disturbance magnitudes. Remarkably similar response trends for the majority of investigated muscles and experimental conditions reinforce the plausibility of the reflex circuits of the model. However, the model's responses lack in amplitude for two experiments with whole body disturbances suggesting that in these cases the proposed reflex circuits need to be amplified by additional control structures such as location-specific cutaneous reflexes. A model that captures these selective amplifications would be able to explain both steady and reactive spinal control of human locomotion. Neuromechanical simulations that investigate hypothesized control models are complementary to gait experiments in better understanding the control of human locomotion.

## 1. Introduction

Understanding the control that underlies human locomotion remains a challenging problem. One reason for this is that many experimental techniques provide only incomplete access to the control circuits, making it impossible to directly probe the entire control involving millions of neurons in complex animals (Vogelstein et al., 2014). Another reason is that the control mechanism seems to vary across species (Orlovskiĭ et al., 1999; Capaday, 2002), which limits our ability to extrapolate control circuits identified with direct methods in other animals to humans (Arshavsky et al., 1985; Zehr and Stein, 1999; Moraud et al., 2016). Yet a third reason is that theoretical results from modeling studies of the control circuitry remain inconclusive (Ijspeert, 2014; Sartori et al., 2016).

Neuromechanical simulations are used as a theoretical tool to study human locomotion control. Since bipedal locomotion emerges from the interaction between the legs and the ground by utilizing and resisting gravitational force (Mochon and McMahon, 1980; McGeer, 1990; Perry and Burnfield, 1992), accounting for the mechanical dynamics as well as the neural control is essential. This integrative approach of simulating the neural control with the biomechanical dynamics allowed researchers to investigate the spinal control layer where a large portion of locomotion control is conducted (Enoka, 2008; Dietz, 2010; Kiehn, 2016). Previously proposed spinal control models range from central pattern generators (CPGs; Aoi et al., 2010) to reflexes (Günther and Ruder, 2003; Geyer and Herr, 2010; Song and Geyer, 2015a) and to a mix of both (Taga et al., 1991; Ogihara and Yamazaki, 2001; Hase and Yamazaki, 2002; Jo and Massaquoi, 2007; Dzeladini et al., 2014). Many of these models with competing control structures are plausible candidates for human control, since they produce locomotion with kinematics, kinetics, or muscle activations similar to the ones observed in humans. Therefore, to genuinely evaluate the plausibility of these models a more in-depth comparison to experimental results is required.

Disturbance reactions provide such a more in-depth comparison. Studying the reaction to disturbances is a common approach to establish system models and to identify controllers (Ogata and Yang, 1970). Specifically for human locomotion, several walking experiments have been conducted that report on the immediate responses of the human spinal control to different types of unexpected disturbances including electrical stimulation (Simonsen and Dyhre-Poulsen, 1999; Courtine et al., 2007), mechanical perturbation at individual leg joints (Dietz et al., 1990; Sinkjaer et al., 1996; Faist et al., 1999), and more natural mechanical perturbation of the whole body (Schillings et al., 1999; Sloot et al., 2015). Although external disturbances have been used in neuromechanical human walking models to either test the robustness of control models (Aoi et al., 2010; Kim et al., 2011; Song and Geyer, 2015a) or to study specific high-level recovery strategies (Jo, 2007; Murai and Yamane, 2011), comparisons of the reference data on the reactions of the human spinal control to the reactions predicted by the different walking models have so far not been performed.

**Figure 1:**
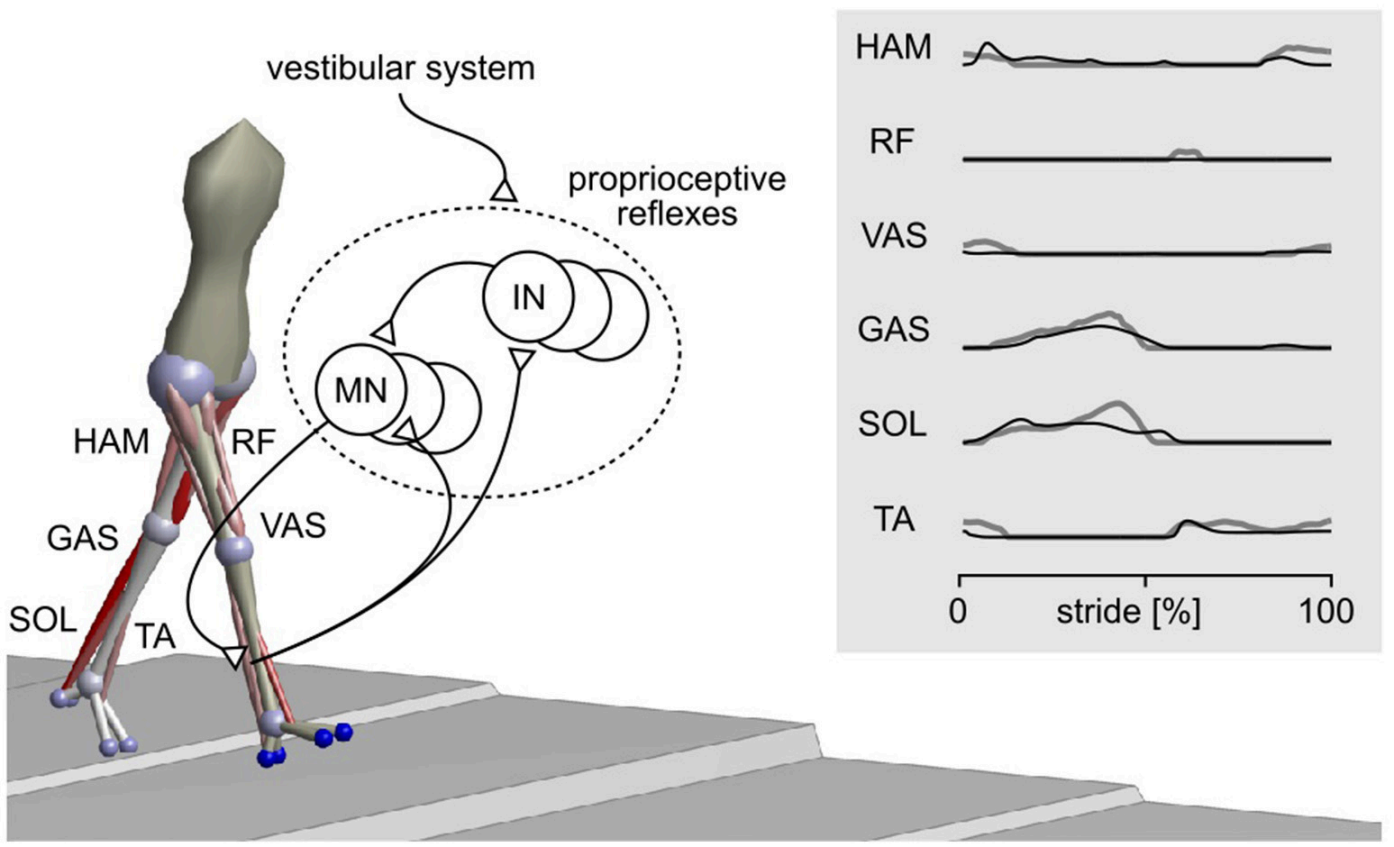
**Spinal reflex-control model of human locomotion**. The sagittal plane components of a 3-D model (Song and Geyer, 2015a) are adopted for the current study. The model mainly uses proprioceptive reflexes to control nine major muscle groups per leg, including hamstrings (HAM), rectus femoris (RF), vasti (VAS), gastrocnemius (GAS), soleus (SOL), and tibialis anterior (TA). The activations of these muscles during normal walking of the model (black lines) and of humans (gray lines) are shown in the right panel. MN, motor neuron; IN, interneuron.

Here, we perform the in-depth comparison of disturbance reactions for one neuromechanical spinal control model of human locomotion (Song and Geyer, 2015a). In previous work, we have shown that this model, which consists of primarily proprioceptive spinal reflexes (Figure 1), can explain undisturbed locomotion behaviors. The model not only produces kinematics, dynamics, and muscle activations similar to humans during normal walking (Figure 1 and Video S1) but also generates other locomotion behaviors such as running, walking on slopes and stairs, and avoiding obstacles. We investigate the plausibility of the model by comparing its reactions against disturbances to those of humans and discuss its implications in better understanding the control of human locomotion.

## 2. Methods

We select a range of unexpected disturbances used in human gait studies from the literature, replicate them in simulation with the neuromechanical model, and compare the models reactions to the reported human experimental data.

### 2.1. Experiment selection

Five disturbance experiments are selected from the literature: electrical stimulation of the lumbar spinal cord to evoke multisegmental monosynaptic responses (MMR; Courtine et al., 2007), mechanical tap of tendons to induce tendon tap reflexes (TR; Dietz et al., 1990; Faist et al., 1999), actuation of the ankle joint to induce stretch reflexes (SR; Sinkjaer et al., 1996), and tripping (TRIP) of the swing leg (Schillings et al., 1999), and slipping (SLIP) of the stance leg (Sloot et al., 2015; refer Video S2 for visual guidance). In these experiments, the reactions of the spinal control are assessed through the changes that occur in the leg muscle activations within a short time after the disturbances. Specifically, the activation changes are measured by surface electromyograms (EMGs) and their trend with respect to gait phase or disturbance magnitude is used to estimate the activity of spinal reflexes.

The five experiments are selected to cover a broad range of disturbances and responses. For instance, from several reports of studies using similar types of disturbances, the ones that include the EMG changes for more leg muscles and across more conditions are selected. Specifically, while both MMR (Courtine et al., 2007) and H-reflex (Capaday and Stein, 1986; Simonsen and Dyhre-Poulsen, 1999) experiments disturb afferent signals using electrical stimulations, the former was selected since MMR disturbs multiple afferents and, as a result, induces responses in more muscles. Similarly, the SR (Sinkjaer et al., 1996), TRIP (Schillings et al., 1999), SLIP (Sloot et al., 2015), and TR (Dietz et al., 1990; Faist et al., 1999) experiments were chosen over similar ones that apply disturbances for fewer conditions (Berger et al., 1984; Yang et al., 1991; Eng et al., 1994; Van de Crommert et al., 1996; Cronin et al., 2009; Chvatal and Ting, 2012; Villarreal et al., 2016). Note that the SLIP experiment by Sloot et al. (2015) reports on muscle responses with latencies of about 150 ms, which are longer than usual for spinal reflexes. Although it is acknowledged that one cannot completely exclude that these responses are long-latency reflexes, we still included the study, as the authors clarify that these apparent latencies are in part an outcome of their experimental protocol for detecting disturbances, and as we could not find an alternative study reporting responses against a range of disturbance intensities. However, to further support our analysis on the response amplitudes in the SLIP experiment (compare Section 3.2), we have verified the consistency of our model results for a similar experiment by Berger et al. (1984), in which the reported responses are clearly within the time window of spinal reflexes.

### 2.2. Replication in simulation

We adapt the original neuromechanical model (Song and Geyer, 2015a) for each of the five experiments (Table 1). Since all the experiments reported on sagittal plane disturbances, the model is first reduced to its sagittal plane musculoskeletal architecture and spinal control. Then, the musculoskeletal properties are scaled (Winter, 2009) to match the average height and weight of the subjects in each experiment (Courtine et al., 2007; Sloot et al., 2015). If this information is not reported (Dietz et al., 1990; Sinkjaer et al., 1996; Faist et al., 1999; Schillings et al., 1999), the height and weight are set to 1.8 m and 80 kg. Finally, the model's control parameters are optimized with the cost function

(1) $$\begin{array}{l} J=C_{E}+c_{v}\|v_{avg}-v_{tgt}\|, \end{array}$$

which encourages energy efficient walking at a target walking speed. In this equation, *C*_*E*_ is the metabolic energy consumed by the muscles, *c*_*v*_ = 100 is a weighting factor, and *v*_*avg*_ and *v*_*tgt*_ are the average and target walking speeds. The target walking speed, *v*_*tgt*_, is set to the reported speed in each experiment. A demonstration of the simulation model can be found in Video S2. More details about the original model and the optimization procedure to obtain control parameters for stable and steady walking are given in (Song and Geyer, 2015a).

**Table 1:** **Experimental setup as described in the human subject studies and as replicated in simulation**.

		**MMR (%)**	**TR (%)**	**SR (%)**	**TRIP**	**SLIP**
Height (m), weight (kg), walking speed (ms^−1^)	$\frac{\text{exp}}{\text{sim}}$	1.75, 64, 0.97	$\frac{-,\;-,\;0.83}{1.8,\;80,\;0.83}$	$\frac{-,\;-,\;0.97}{1.8,\;80,\;0.97}$	$\frac{-,\;-,\;1.11}{1.8,\;80,\;1.11}$	$\frac{\text{BMI }=\;23,\;1.20}{1.8,\;75,\;1.20}$
Disturbance	$\frac{\text{exp}}{\text{sim}}$	$\frac{\begin{array}{c} 1\text{ms electrical square pulse}\\ \text{ percutaneously at lumbar spinal cord} \end{array}}{10\text{ms square pulse at afferent signals}}$	$\frac{\text{Tendon tap with }90\text{g},\;1.5\text{m}{\text{s}}^{-1}\text{ hammer}}{\text{Muscle length change induced by hammer tap}}$	Ankle flexion of 8° with velocity of 250°s^−1^	2.2 kg obstacle bumped by swing leg	Speed change of split-belt treadmill at 150 ms after heel strike
conditions	$\frac{\text{exp}}{\text{sim}}$	16 equal phases over stride	$\frac{\text{Various phases over stride}}{16\text{ equal phases over stride}}$	8 equal phases over stride	Various phases over 5~75% of swing	Speed changes of 0.1 to 0.5 with increments of 0.1 ms^−1^

*exp: human experiment; sim: simulation replication*.

The disturbances were simulated for the reported conditions in each experiment, which either included different gait phases (for MMR, TR, SR, and TRIP) or different disturbance intensities (for SLIP). The mechanical disturbances of the SR, TRIP, and SLIP experiments were directly replicated in the simulation by modeling an unexpected ankle flexion, the encounter of the tripping obstacle, and the shift of the supporting ground with the same parameters as reported in each experiment, respectively.

The MMR and TR experiments were less straightforward to replicate in simulation, as the neuromechanical model does not include the corresponding physiological detail. In the MMR experiment (Courtine et al., 2007), muscle responses (spikes with about 20 ms durations) are induced by percutaneous electrical stimulation (1 ms square pulses) at the lumbar spinal cord, which disturbs the afferent pathways from the legs. Instead of modeling the electrophysiological dynamics such as the filtering effects of the skin layer, the MMR disturbance was simulated as 10 ms square pulses that were simultaneously added to the afferent signals from all muscles. The duration of 10 ms was chosen because it created similar muscle responses (spikes with about 20 ms durations) in the model. The amplitudes of the square pulses were set to be arbitrarily large (maximum isometric forces, *F*_*m*_, for force afferents; optimum length, *l*_*ce*_, for length afferents; and maximum-contraction-velocity value, |*v*_*max*_|, for velocity afferents) to evoke responses much larger than the normal activations seen during walking, as reported in the MMR experiment (Courtine et al., 2007).

For the TR experiment, it is generally observed that the tendon tap reflex amplitude is proportional to the tapping force (Mildren et al., 2016), although the neurophysiological process behind this observation is not well understood (Zhang et al., 1999). The effect of tendon taps was modeled by simulating the length changes in the muscle tendon unit affected by the tapping. Specifically, we simulated the length change based on the tension of the muscle and the kinetic energy of the tapping hammer. As a result, the effect of the taps on length change varied over the gait cycle according to the variation of the muscle tension.

### 2.3. Reaction comparisons

**Figure 2:**
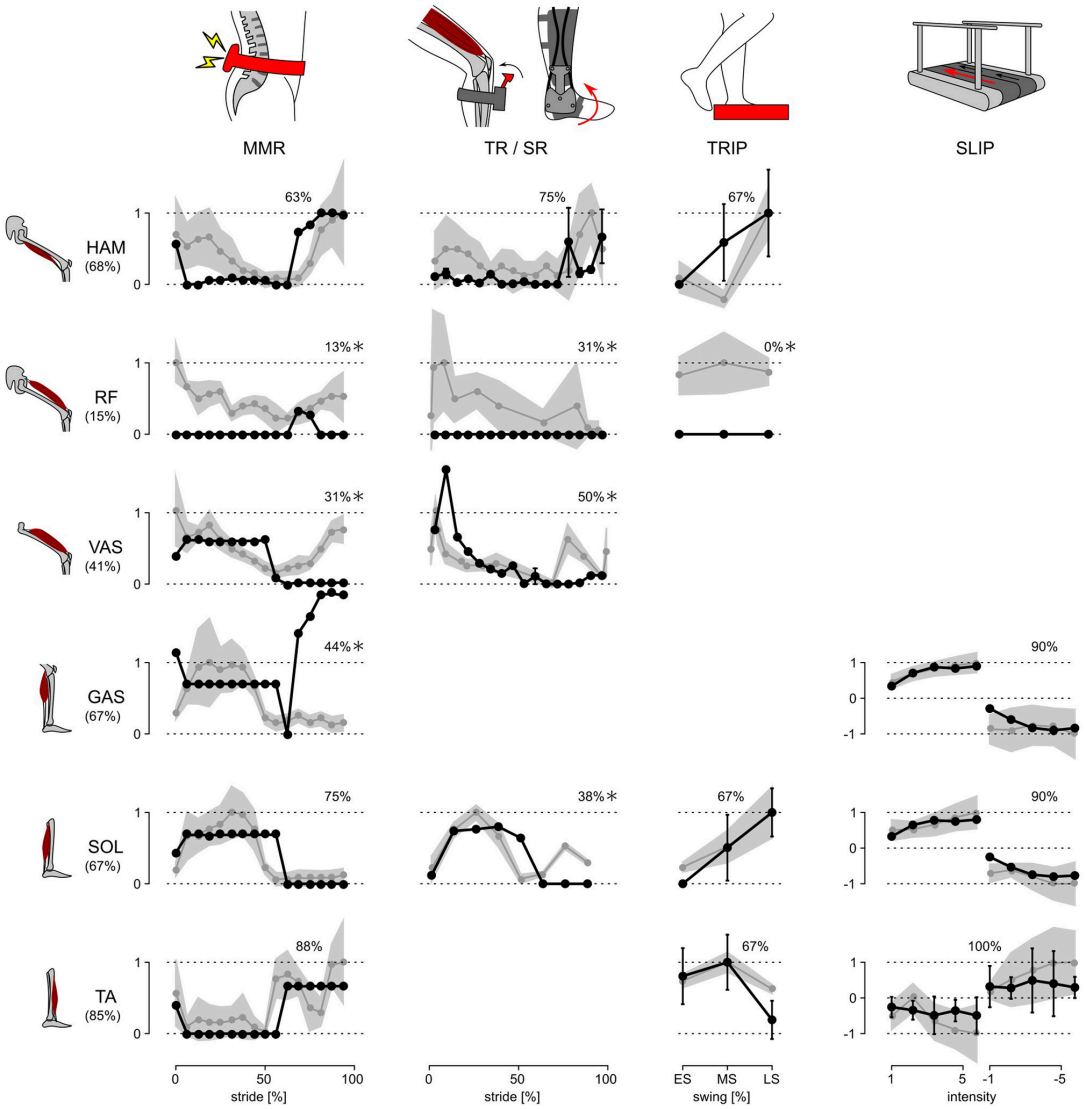
**Response trends**. The responses of the model and human subjects in all five disturbance experiments are shown. Human responses (gray lines) are normalized with respect to their maximum value in each experiment and for each muscle. The model responses (black lines) are linearly scaled to place as many of the responses as possible within ±1 *s.d*. of the human responses (gray shaded area). The % of the model responses within ±1 *s.d*. of human responses are shown at the top of each graph, and those which are ≤50% are marked with ^*^.

The response trends and amplitudes were compared separately for each experiment and muscle. While the model has nine muscles per leg, data for only six muscles was available in the literature (compare Figure 1). Similarities of the response trends were quantified as the % of the model responses that lie within ±1 standard deviation (*s.d*.) of human responses when linearly scaled to maximize overlap. For example, 12 out of 16 of the model's SOL responses in the MMR experiment lie within ±1 *s.d*. of the corresponding human responses and thus the similarity is 12/16 = 75% (Figure 2).

The response amplitudes are only compared for the SR, TRIP, and SLIP experiments. The MMR and TR disturbances induce synchronous and artificially exaggerated muscle activation responses, which is not observed in normal voluntary activations (Yang et al., 1991). As the model does not include these artificially synchronized muscle activations, the response amplitudes are not meaningful to compare for these studies.

## 3. Results

### 3.1. Response trends

The neuromechanical control model and humans react to disturbances with a similar trend for the majority of investigated muscles and experimental conditions. Figure 2 summarizes the changes in muscle activation organized by disturbance experiment and leg muscle. The changes observed in humans (gray lines and shaded areas indicating ±1 *s.d*.) are normalized to their peak value and overlaid by the corresponding changes of the model (scaled to maximize overlap and compare trends as described in Section 2.3, black lines). While some of the response trends do not match well (≤50% overlap within one *s.d*., comparisons marked with *), for the majority of the investigated muscles and experimental conditions the scaled model responses lie within one *s.d*. of the human responses (78% average overlap for unmarked comparisons).

For several of the marked comparisons, simple modifications of either the reflex control or the model tuning could improve the overlap. First, in the model, the rectus femoris muscle (RF) is used mainly for sensing but not actuation. As a result, it cannot change activation except during swing. In the human experiments, by contrast, RF shows response trends similar to the synergistic vasti muscle group (VAS) throughout stride, although careful interpretation of these RF responses is needed, since surface EMGs of RF, which are used in the disturbance experiments, are prone to crosstalk from VAS (Nene et al., 2004). If fine wire EMG of RF reveal response trends similar to those of VAS, these trends can be reproduced by modifying the model to control RF with the same reflex pathways as VAS. Such modification is tenable in the functional point of view, since RF and VAS share a common role of knee extension.

Second, the difference between human and model responses of the vasti and the gastrocnemius muscles (GAS) during late swing may be an artifact of the model tuning process, which only considered undisturbed walking. The late swing reflexes that control VAS and GAS in the model do not engage during undisturbed locomotion (Song and Geyer, 2015a), and thus the optimization process sets their parameters to arbitrary values as far as they do not effect normal walking. In other words, these control parameters could be further tuned to improve the overlap with human responses for the two muscles without changing the undisturbed walking behavior.

Finally, the weak overlap for the soleus muscle (SOL) in the late swing phase of the SR experiment may be the result of natural variability in humans. It is known from human experiments using the H-reflex, the electrically elicited equivalent of the stretch reflex, that the swing phase responses in SOL vary among subjects between no responses (similar to the trend predicted by the model) and the responses shown in the SR experiment (Simonsen and Dyhre-Poulsen, 1999).

### 3.2. Response amplitudes

Whereas, the model captures the majority of the human response trends, it clearly underestimates the response amplitudes for the more natural, whole body disturbances. In the SR experiments, the model reacts with amplitudes in the muscle activation changes that are similar to the ones reported for humans (about 90% of human amplitudes). Yet in the more natural TRIP and SLIP experiments, the response amplitudes are very small in the model (about 20 and 4%, respectively, and 8% for the experiment in Berger et al., 1984 as noted in Section 2.1). The difference occurs as the reflexes of the model only respond to changes in the muscle lengths, velocities and forces, and the SR disturbance induces much larger changes (up to about 100 times) in these proprioceptive signals than the TRIP and SLIP disturbances, which act on the muscles through the entire body and its mechanical inertia.

**Figure 3:**
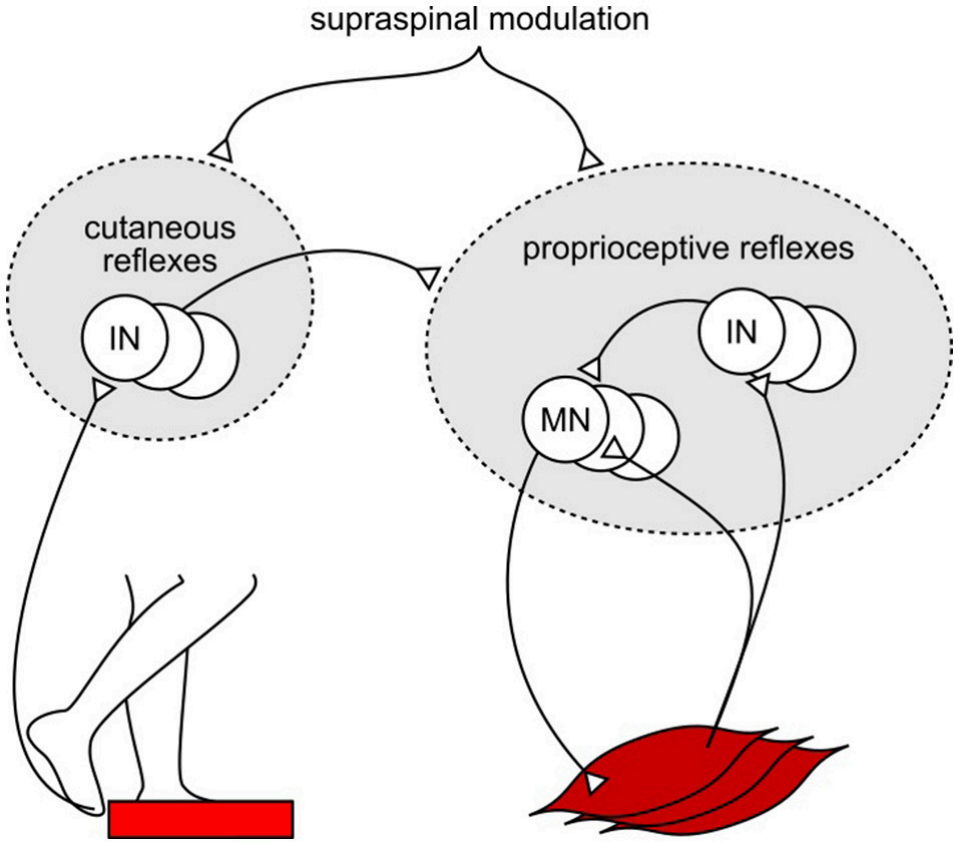
**Example of proposed cutaneous amplification of proprioceptive reflex control during tripping**. Location specific skin sensors at the foot detect an obstacle encounter. Cutaneous reflex pathways return this information to the spinal cord and amplify the proprioceptive reflex control of locomotion.

One explanation for the shortfall in the model's response amplitudes could be the missing integration of reflex pathways from skin receptors. Experimental studies have shown that cutaneous reflexes evoke muscle responses with different trends across the gait cycle depending on the location of the skin receptors (Van Wezel et al., 1997; Duysens et al., 2000; Nakajima et al., 2016). Additional modulation of the model's current proprioceptive reflexes by location-specific cutaneous reflexes (Figure 3), which have been observed in cat experiments (Lundberg et al., 1987), could produce human-like muscle response amplitudes in all experiments without altering the response trends. Such additional modulation against specific disturbances, such as those in SLIP and TRIP experiments, is also in agreement with previous observations that cutaneous stimulations are not accountable for the responses against certain joint specific disturbances (for example, in SR experiment; Grey et al., 2001) but do evoke muscle responses during human walking (Nakajima et al., 2016). However, the functional relevance of this amplification remains open for speculation. For instance, it could promote the recovery strategies seen during human tripping (elevating and lowering strategies in early and late swing; Eng et al., 1994) and slipping (ankle and hip strategies for anterior-posterior and medial-lateral perturbations; Oliveira et al., 2012).

## 4. Discussion

A neuromechanical model of human locomotion has been evaluated by comparing its reactions to disturbances with those of humans during walking. The comparison of the response trends reinforces the plausibility of the majority of the model's reflex circuits. However, the observation of smaller response amplitudes of the model for the whole body disturbances suggests that these circuits are selectively amplified in humans.

**Figure 4:**
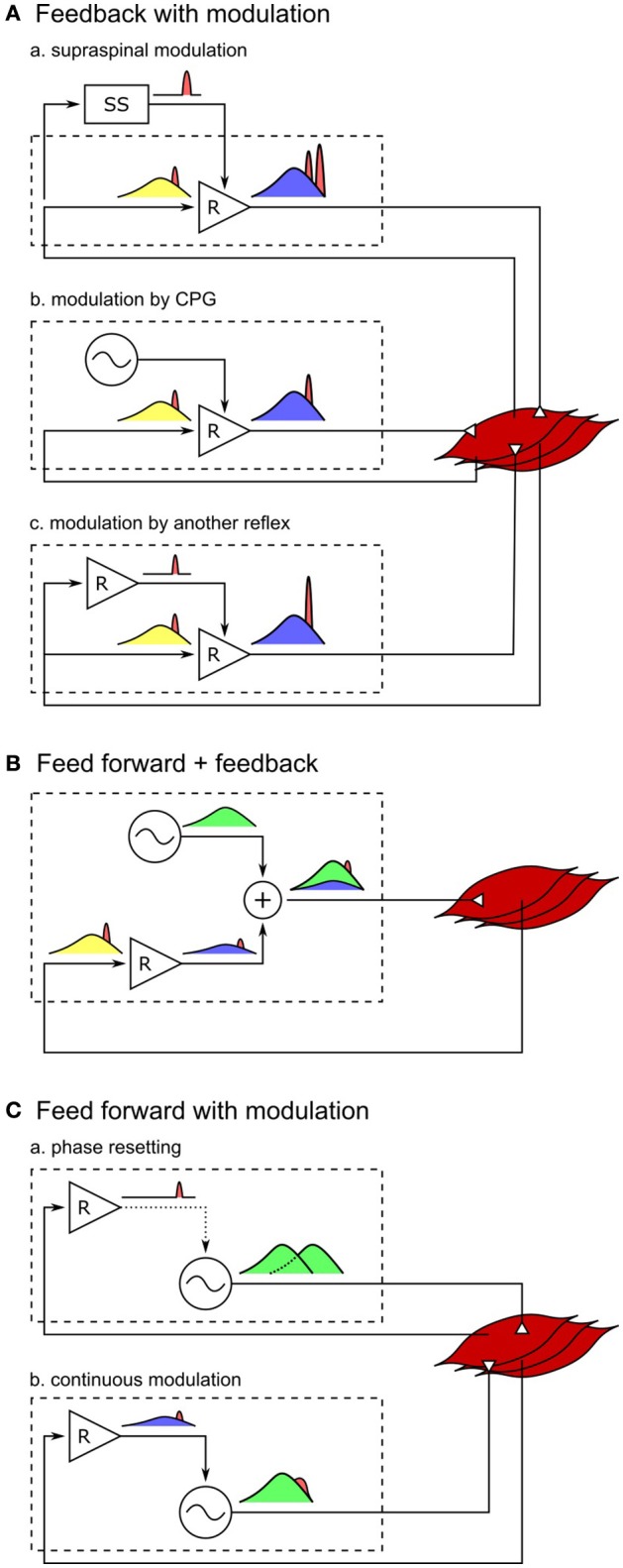
**Spinal control hypotheses of the generation of muscle activations**. Each block diagram represents a spinal mechanism of generating muscle activations, where the spinal control can potentially consist of serial and parallel combinations of the each mechanism. Outputs of reflex circuits and CPGs are marked in blue and green, respectively, afferent signals during normal walking are marked in yellow, and those signals in response to disturbances are marked in red. **(A-a)** Responses through the supraspinal system appears with larger time delays than the spinal responses. This holds true for supraspinal modulations of any spinal control (not shown for **B,C**). **(A-b)** Modulation of reflex circuits by pure CPGs does not change the responsive activations. **(A-c)** Response activations of reflexes can be selectively modulated by additional reflex circuits. **(B)** If muscle activations are generated mostly by CPGs, in other words, if the reflex circuits generate only a small portion of the activation signals, the response to the change in afferent signals would be small as well. **(C-a)** Phase resetting of CPGs results in persistent phase shift of the muscle activation signals. **(C-b)** If CPGs are continuously modulated by sensory feedback, all afferent signals, including the disturbance signals, get modulated by CPG dynamics.

An extension of the current control model with additional circuits that modulate the current reflex gains would likely be able to better reproduce both the human response trends and amplitudes (Figure 4A). For example, instead of the abrupt switches in the reflex gains in the current model, either the supraspinal control (Jo and Massaquoi, 2007; Song and Geyer, 2015a) or CPGs can gradually change these reflex gains (Figures 4A-a,b) and shape the response trends closer to humans (for example, during the transitions between stance and swing phases in VAS, GAS, and SOL, Figure 2). In addition, selective amplifications of response amplitudes for particular disturbances can be realized through additional reflex pathways that modulate the reflex gains based on the detection of those particular disturbances (Figure 4A-c). These additional reflex gain modulations would be able to reproduce the human control during steady walking as well as its reactions against unexpected disturbances.

On the other hand, it remains open whether other types of models, where CPGs generate motor outputs, can reproduce steady and reactive human walking behaviors with a similar level of agreement. It is often hypothesized that CPGs generate some portion or most of the normal (background) muscle activations while reflexes in parallel generate the remaining portion (Duysens and Van de Crommert, 1998; Dominici et al., 2011; Kiehn, 2016; Figure 4B). However, it is less likely that the previously proposed human walking models based on this hypothesis (Ogihara and Yamazaki, 2001; Jo and Massaquoi, 2007; Dzeladini et al., 2014) can explain human responses observed in the disturbance experiments, because the more of the normal activations is generated in a feed-forward manner by CPGs the smaller the response amplitudes will be, which stands in contrast to the large reactions observed in humans. For example, in a model that generates 90% of the normal activations with CPGs and the remaining 10% with the reflex pathways of the reflex-based model (Dzeladini et al., 2014), the response trends will remain the same but the response amplitudes will only be a tenth of the reflex-based model. Alternatively, the responsive activations could also be partially generated by CPGs as they get modulated by sensory feedback (Figure 4C). For example, phase shifts in CPG activations in response to perturbations, which is called phase resetting (Figure 4C-a), have been observed in cats (Conway et al., 1987; Schomburg et al., 1998) and have been proposed to increase the robustness of human walking (Yamasaki et al., 2003; Aoi et al., 2010). However, the responses observed in the disturbance experiments considered in this study do not seem to originate from phase resetting of CPGs since they are transient responses rather than persistent phase shifts. Finally, CPGs have also been proposed to be continuously modulated by sensory feedback in many models, where the muscle responses result from more complicated CPG dynamics (Taga et al., 1991; Ogihara and Yamazaki, 2001; Hase and Yamazaki, 2002; McCrea and Rybak, 2008; Figure 4C-b). CPGs are usually modeled to consist of mutually inhibiting neurons with internal dynamics (Matsuoka, 1985), and many human walking models (Taga et al., 1991; Ogihara and Yamazaki, 2001; Hase and Yamazaki, 2002) incorporate continuous sensory feedback modulation of CPGs by adding afferent signals to this internal dynamics (for example, in the form of $\tau \dot{u}=-u+\mathrm{other}\text{-}\mathrm{terms}+\mathrm{feedback}$, where τ is a time constant and *u* is the neural output). In this case, the muscle responses are likely to be slower and smaller, since the disturbance signals need to be integrated to appear in the neural outputs of the CPGs. Therefore, in order to explain both steady and reactive behaviors during human walking with control structures in which CPGs generate muscle activations, more complicated reflex circuits may be necessary that selectively amplify the responses not only for the whole body disturbances but also for the other disturbances.

Still, there is clear evidence that CPGs are highly involved in locomotion of many animals including mammals, and it is reasonable to expect human locomotion involves a similar control structure if the functional role of CPGs remained valid in the course of evolution to upright bipedal locomotion (Capaday, 2002; MacKay-Lyons, 2002; Ijspeert, 2008). One functional role that has been proposed to be realized by CPGs is the generation of transitional behaviors such as changing gait, as well as locomotion speed and direction. This view is supported by observations on decerebrate animals, where simple supraspinal stimulations control locomotion by modulating the frequency and amplitude of CPGs (Armstrong, 1988; Stein et al., 1997; Sirota et al., 2000). It has been shown with a neuromechanical model that human locomotion speed can be controlled in a similar way by modulating CPGs of the hip muscles (Van der Noot et al., 2015). On the other hand, transitional behaviors including speed and directional changes also can be realized in the absence of CPGs by changing the reflex gains directly through the supraspinal control (Song and Geyer, 2012, 2015a,b). Therefore, the role of CPGs in transitional locomotion behaviors of humans calls for further experimental studies. To this end, investigating the responses of the hip muscles (Hof and Duysens, 2013), which lack in previous gait disturbance experiments, can be crucial.

Our results also show that solely relying on indirect experimental observations can be misleading when assessing the role of reflexes. First, the changes in muscle responses do not necessarily indicate modulation of reflex gains. For example, in the TR experiment the changes in the model's HAM and VAS responses during stance (Figure 2) result from the changes in muscle configurations while the reflex gains remain constant. Second, the correlation between the muscle states and muscle responses is not sufficient to explain the underlying muscle reflexes. For instance, in a gait experiment similar to the SR experiment, Yang et al. (1991) suggested velocity feedback to contribute about 45% in the generation of SOL activations during the stance phase. The suggested contribution is based on the correlation between the changes in ankle velocity and the responses in SOL activation. However, as noted by the authors of the study, this quantification neglects the potential contributions of different afferent pathways. Performing the same correlation-based analysis in our model suggests a contribution of about 40% of velocity feedback in the stance control of SOL, even though the model uses no velocity feedback but 100% force feedback.

Although, the findings of our study may help to construct a model that can explain the steady and reactive spinal control of human walking, it will take further research to settle the actual circuitries in humans. First, neuromechanical simulations with more physiological details will be needed to incorporate other types of experimental studies in the evaluation of control models. For instance, we would be able to compare the response amplitudes of our control model to human responses in MMR and TR experiments if our simulations could more faithfully describe the relationship between cutaneous electrical stimulation and synchronous muscle activation as well as the related neurophysiology. Second, other models which can explain normal human walking should also be subjected to gait disturbance experiments to genuinely evaluate their plausibility and arrive at a consensus about what the human circuitry might be. Finally, the resulting control model should be verified by direct probing of the proposed neural circuits in human experiments. Although, it is currently impossible to probe the entire control of humans that involves millions of neurons, a control model that is thoroughly evaluated and specified may substantially reduce the search space. Evaluation beyond steady behavior will play an important role in this quest.

## Author contributions

SS and HG designed the research. SS developed the computational model, conducted the simulation studies, and analyzed the data. SS and HG drafted the manuscript and approved the final version of the manuscript.

## Funding

This work is supported in part by the National Science Foundation (grant no. 1527140), and the Richard King Mellon Foundation Presidential Fellowship in the Life Sciences at Carnegie Mellon University.

### Conflict of interest statement

The authors declare that the research was conducted in the absence of any commercial or financial relationships that could be construed as a potential conflict of interest.

## Supplementary material

Video S1 **Spinal-reflex-based neuromuscular model of human locomotion**.

Video S2 **Simulation replications of the human disturbance experiments**.
